# Supplementary material for: Crocosphaera as a Major Consumer of Fixed Nitrogen
Source: Microbiol Spectr. 2022 Jun 30;10(4):e02177-21. doi: 10.1128/spectrum.02177-21 (PMC9431459; doi:10.1128/spectrum.02177-21)
Supplement: Supplemental file 1 — Supplemental material. Download spectrum.02177-21-s0001.pdf, PDF file, 2.4 MB [file spectrum.02177-21-s0001.pdf]

Table S1 The initial physical and chemical conditions of seawater collected for each bioassay experiment. For all data, means are shown with ± standard deviation for triplicate samples. ND, no data. SRP; soluble reactive phosphorus, TFe; total iron, DFe; dissolved iron (< 0.22 µm), *Pro.*; *Prochlorococcus*, *Syn.*; *Synechococcus*, *Cro.*; *Crocosphaera*, PicoE; Pico-eukaryotes.

Exp.	Date In 2008	Chl *a* (ng L^{-1})	Salinity	Temperature (ºC)	NO_3^-+NO_2^- (nM)	NH_4^+ (nM)	SRP (nM)	TFe (nM)	DFe (nM)	*Pro.* (cells mL^{-1})	*Syn.* (cells mL^{-1})	*Cro.* (cells mL^{-1})	PicoE. (cells mL^{-1})
M1	6 June	24	34.27	29.0	<3	<3	56	0.34	0.11	5275 ± 1628	70 ± 5	32 ± 62	366 ± 118
M2	10 June	25	34.19	29.3	<3	<3	51	0.29	0.17	2235 ± 226	38 ± 3	129	614 ± 72
M3	14 June	28	34.24	29.1	<3	9	64	ND	ND	3609 ± 1051	35 ± 29	126 ± 32	410 ± 137
M4	18 June	61	34.13	29.1	<3	15	31	0.47	0.44	26212 ± 2227	1280 ± 131	1513 ± 684	804 ± 44
M5	22 June	98	33.94	28.9	7	36	31	1.01	0.16	26767 ± 3662	522 ± 101	306 ± 112	553 ± 54

Table S2. *In situ* nitrogen fixation rate at 10 m depth. For all data, means are shown with ± standard deviation for triplicate samples.

Ex.	Date In 2008	*In situ* N_2 fixation rate (nmolN $L^{-1}d^{-1}$)	*In situ* N_2 fixation rate <10µm (nmolN $L^{-1}d^{-1}$)
M1	6 June	1.33 ± 1.81	2.75 ± 4.68
M2	10 June	2.37 ± 0.59	0.66 ± 0.96
M3	14 June	0.19 ± 2.06	0.28 ± 2.40
M4	18 June	6.65 ± 2.52	2.37 ± 0.77
M5	22 June	4.94 ± 1.38	4.75 ± 1.72

Table S3. The nutrient concentration of initial sample collected for each bioassay experiment (this is after filtration, before incubation). For all data, means are shown with ± standard deviation for triplicate samples. SRP; soluble reactive phosphorus.

Ex.	NO_2^-+NO_3^- (nM)	NH_4^+ (nM)	SRP (nM)
M1	24 ± 3	<3	54 ± 3
M2	4 ± 5	<3	44 ± 1
M3	<3	15 ± 1	64 ± 0
M4	<3	<3	22 ± 0
M5	4 ± 0	34 ± 3	29 ± 1

Table S4. Values used for parameters

Parameter	Unit	Value
m_i	d^{-1}	0.4
For NH_4^+ added case		
N_{Cro}	nmol L^{-1}	2.50*
N_{Oth}	nmol L^{-1}	5.00*
$V_{Max,Cro}^{NH4}$	d^{-1}	6.6
$V_{Max,Cro}^{NO3}$	d^{-1}	2.8
$V_{Max,Oth}^{NH4}$	d^{-1}	1.1
$V_{Max,Oth}^{NO3}$	d^{-1}	1.8
K_{Cro}^{NH4}	nmol L^{-1}	140
K_{Cro}^{NO3}	nmol L^{-1}	80
K_{Oth}^{NH4}	nmol L^{-1}	6
K_{Oth}^{NO3}	nmol L^{-1}	500
For NO_3^- added case		
N_{Cro}	nmol L^{-1}	4.36*
N_{Oth}	nmol L^{-1}	5.83*s
$V_{Max,Cro}^{NH4}$	d^{-1}	8
$V_{Max,Cro}^{NO3}$	d^{-1}	1.3
$V_{Max,Oth}^{NH4}$	d^{-1}	0.9
$V_{Max,Oth}^{NO3}$	d^{-1}	0.5
K_{Cro}^{NH4}	nmol L^{-1}	70
K_{Cro}^{NO3}	nmol L^{-1}	90
K_{Oth}^{NH4}	nmol L^{-1}	2
K_{Oth}^{NO3}	nmol L^{-1}	700
Ecosystem simulation		
N_{Cro}	nmol L^{-1}	1000*
N_{Oth}	nmol L^{-1}	1000*
N_{Zoo}	nmol L^{-1}	1000*
m_{Zoo}	d^{-2}	0.01#
G_{max}	d^{-1}	7.5
K_G	d^{-1}	500#

* Initial value. #Value from Inomura et al. (1).

Table S5. degrees of freedom (df), *F*-values and *p*-values of the two-way RM-ANOVA for cell density. Day represents the timing of harvest (1, 2, 3); Treatment represents the enriched nutrient (Cont, PO_4^{3-}, NO_3^-, NH_4^+, Urea).

		df	*F*-value	*p*-value
Exp.			*Crocosphaera*	
M1	Day	2	5.163	0.01<*p*<0.05
	Treatment	4	0.832	>0.05
	Day × Treatment	8	1.474	>0.05
M2	Day	2	48.039	<0.01
	Treatment	4	1.527	>0.05
	Day × Treatment	8	1.316	>0.05
M3	Day	2	66.073	<0.01
	Treatment	4	1.612	>0.05
	Day × Treatment	8	1.347	>0.05
M4	Day	2	59.354	<0.01
	Treatment	4	9.530	<0.01
	Day × Treatment	8	1.688	>0.05
M5	Day	2	26.157	<0.01
	Treatment	4	1.353	>0.05
	Day × Treatment	8	1.269	>0.05

11

Table S6. Summary of the effects of nutrient additions on cell density and cell size of *Crocosphaera* in each bioassay experiment. Cell density and cell size were measured from separate triplicate bottles. The response of the phytoplankton communities to the different treatments was compared by post hoc Tukey test, to compare the means between five treatments (n = 3 replicates per treatment throughout, degrees of freedom = 40). Significant differences ($p < 0.05$) between individual treatments are indicated by <; = indicates no significant differences. Cont, PO4, NO3, NH4, Urea refer to the treatment of control, PO_4^{3+}, NO_3^-, NH_4^+ and urea respectively.

Exp.	Day	Cell density	Cell size
M1	1	Cont = PO4 = NO3 = NH4 = Urea	Cont = PO4 = NO3 = NH4 = Urea
	2	Cont = PO4 = NO3 = NH4 = Urea	Cont = PO4 = NO3 = NH4 = Urea
	3	Cont = PO4 = NO3 = NH4 = Urea	Cont = PO4 = NO3 = NH4 = Urea
M2	1	Cont = PO4 = NO3 = NH4 = Urea	Cont = PO4 = NO3 = NH4 = Urea
	2	Cont = PO4 = NO3 = NH4 = Urea	NO3 = NH4 = Urea < PO4 < Cont
	3	Cont = PO4 = NO3 = NH4 = Urea	Cont = PO4 = NO3 = NH4 = Urea
M3	1	Cont = PO4 = NO3 = NH4 = Urea	Cont = PO4 = NO3 = NH4 < Urea
	2	Cont = PO4 = NO3 = NH4 = Urea	Cont = PO4 = NO3 = NH4 = Urea
	3	Cont = PO4 = NO3 = NH4 = Urea	Cont = PO4 = NO3 = NH4 = Urea
M4	1	Cont = PO4 = NO3 = NH4 = Urea	PO4 < NO3 = NH4 = Urea < Cont
	2	Cont = PO4 = NO3 = NH4 = Urea	PO4 < Urea < Cont = NO3 = NH4
	3	Cont = PO4 = NO3 = NH4 = Urea	PO4 < NO3 = Urea < Cont = NH4
M5	1	Cont = PO4 = NO3 = NH4 = Urea	Cont = PO4 = NO3 = NH4 = Urea
	2	Cont = PO4 = NO3 = NH4 = Urea	PO4 = NO3 = Urea < NH4 < Cont
	3	Cont = PO4 = NO3 = NH4 = Urea	Cont = PO4 = NO3 = NH4 = Urea

12

Table S7. Procedure of macro-nutrient bioassays (M1-M5). Seawater was first pre-filtered through 1 μm in-line cartridge filter (Micropore EU, ORGANO) and dispensed into triplicate 4L poly carbonate bottles, which are cleaned by neutral detergent and 0.3 N HCl. Bottles were incubated on-deck in flow through seawater tanks covered with neutral density screen to attenuate light intensity to 50% if its corresponding surface value. Samples were harvested after 1, 2, 3 days of incubation. Non; no enrichment, -; not expected.

Treatment	Enrichment	Final concentration (nM)
Control	Non	-
PO_4^{3-}	NaH_2PO_4	10 nM-P
NO_3^-	$NaNO_3$	100 nM-N
NH_4^+	NH_4Cl	100 nM-N
Urea	$CO(NH_2)_2$	100 nM-N

Table S8. Procedure of Fe addition bioassays (Fe1-3). Seawater was first pre-filtered through 10 µm in-line cartridge filter (Micropore EU, ORGANO) and dispensed into 2L poly carbonate bottles (duplicate for Exp. Fe1, triplicate for Exps. Fe2 and Fe3), which are cleaned according to ref. 65. Bottles were incubated on-deck in flow through seawater tanks covered with neutral density screen to attenuate light intensity to 50% if its corresponding surface value. Samples were harvested after 1 and 3 days of incubation. Non; no enrichment, -; not expected.

Treatment	Enrichment	Final concentration
Control	Non	-
Fe	$FeCl_3$	1 nM-Fe
PO_4^{3-}	NaH_2PO_4	10 nM-P
Fe + PO_4^{3-}	$FeCl_3$ + NaH_2PO_4	1 nM-Fe + 10 nM-P
Fe + NO_3^-	$FeCl_3$ + $NaNO_3$	1 nM-Fe + 100 nM-N

Table S9. Used symboles, units and definitions in the quantitative model

Symbol	Unit	Definition
i	n.a.	i = Cro, Oth
j	n.a.	$j = NO_3^-, NH_4^+$
N_i	nmol L^{-1}	Cellular nitrogen concentration of phytoplankton i per volume water
t	d	Time
μ_i	d^{-1}	Growth rate of phytoplankton i
m_i	d^{-1}	Mortality rate of phytoplankton i
$V_{Max,i}^{j}$	d^{-1}	Maximum uptake rate of nutrient j by phytoplankton i
$[j]$	nmol L^{-1}	Concentration of nutrient j
K_i^j	nmol L^{-1}	Half saturation constant of nutrient j
G_i	d^{-1}	Grazing rate of phytoplankton i
N_{Zoo}	nmol L^{-1}	Nitrogen concentration in zooplankton per volume water
m_{Zoo}	d^{-2}	Quadratic mortality rate of zooplankton
G_{max}	d^{-1}	Maximum grazing rate
K_G	nmol L^{-1}	Grazing half saturation

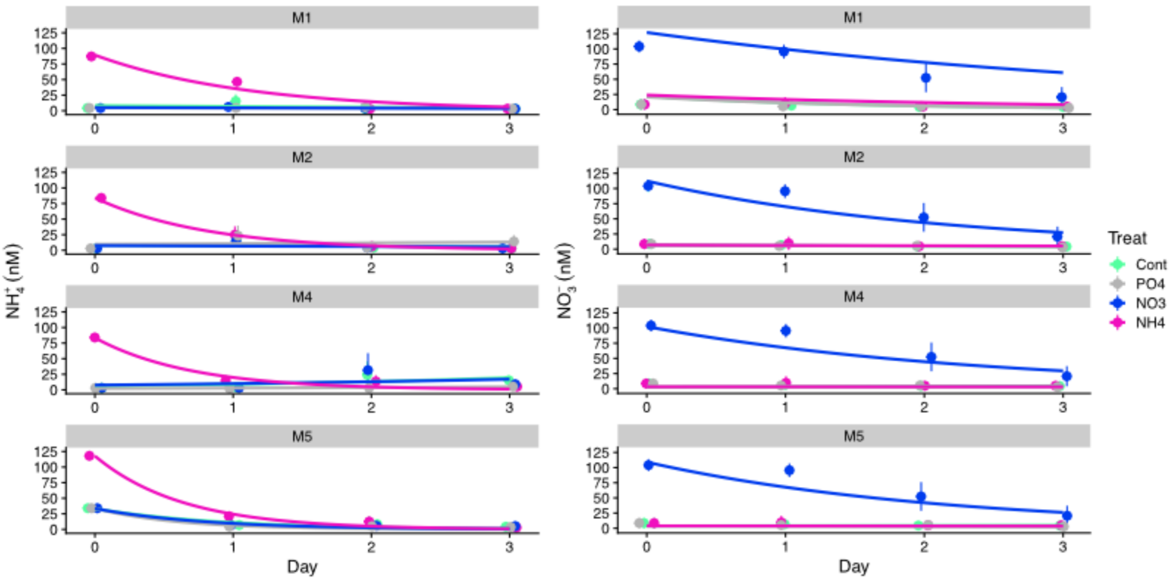

Fig. S1. Temporal change in NH_4^+ and NO_3^- concentrations of Ex. M1, M2, M4 and M5. (A) NH_4^+ concentration in the NH_4^+ treatment exponentially decreased during the experiment down to the detection limit of 6 nM on day 3. (B) NO_3^- concentrations in the NO_3^- treatment exponentially decreased during the experiment but enriched NO_3^- was not always entirely consumed. Error bar shows a standard deviation of triplicate.

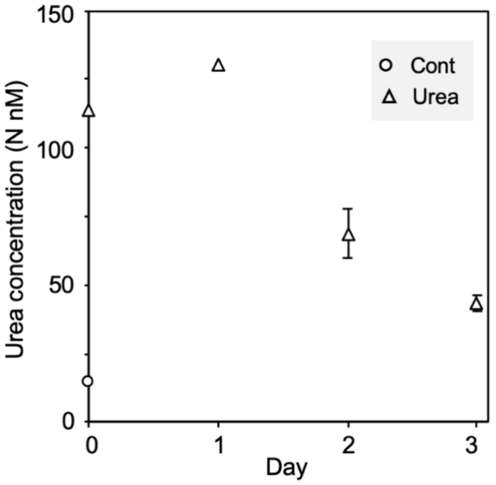

Fig. S2 Temporal change in Urea-N concentration. Concentration in control was measured only at the initial. Error bar shows the standard deviation of triplicate samples.

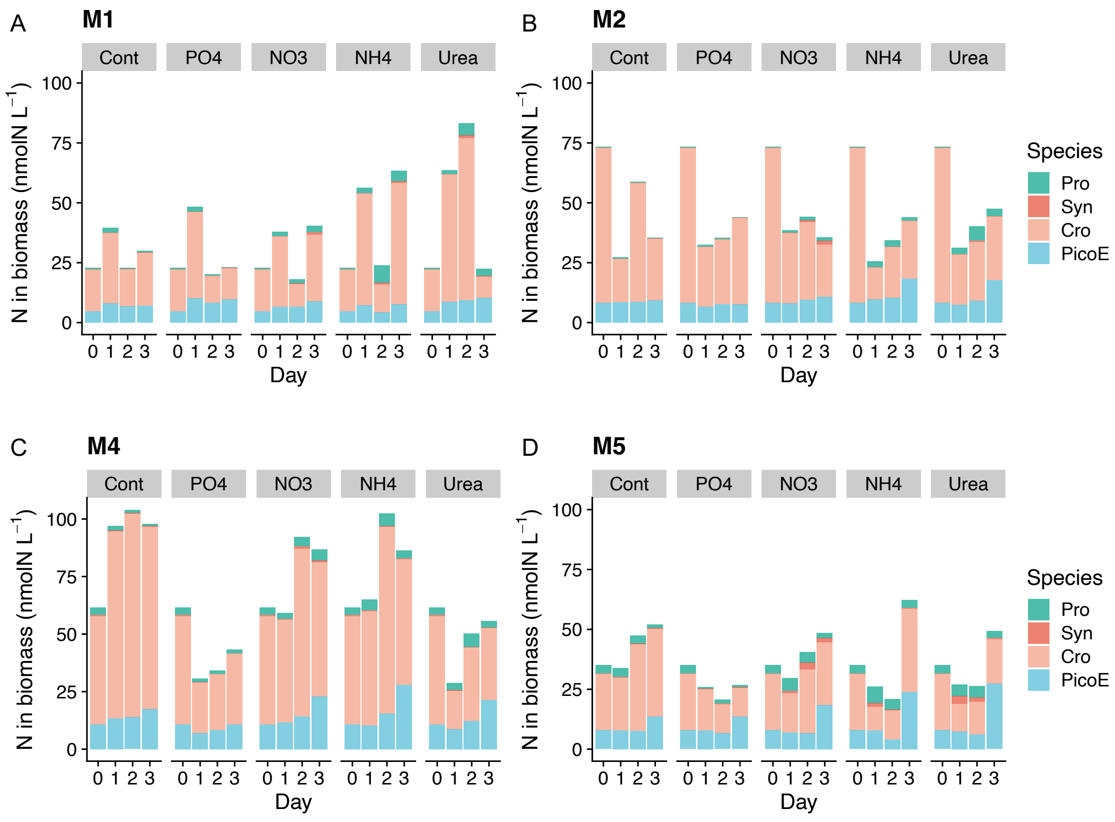

Fig. S3 N in biomass in each treatment and its contribution of each phytoplankton group of experiment M1, M2, M4 and M5. Pro; *Prochlorococcus*, Syn; *Synechococcus,* Cro; *Crocosphaera*, PicoE; pico-eukaryotes.

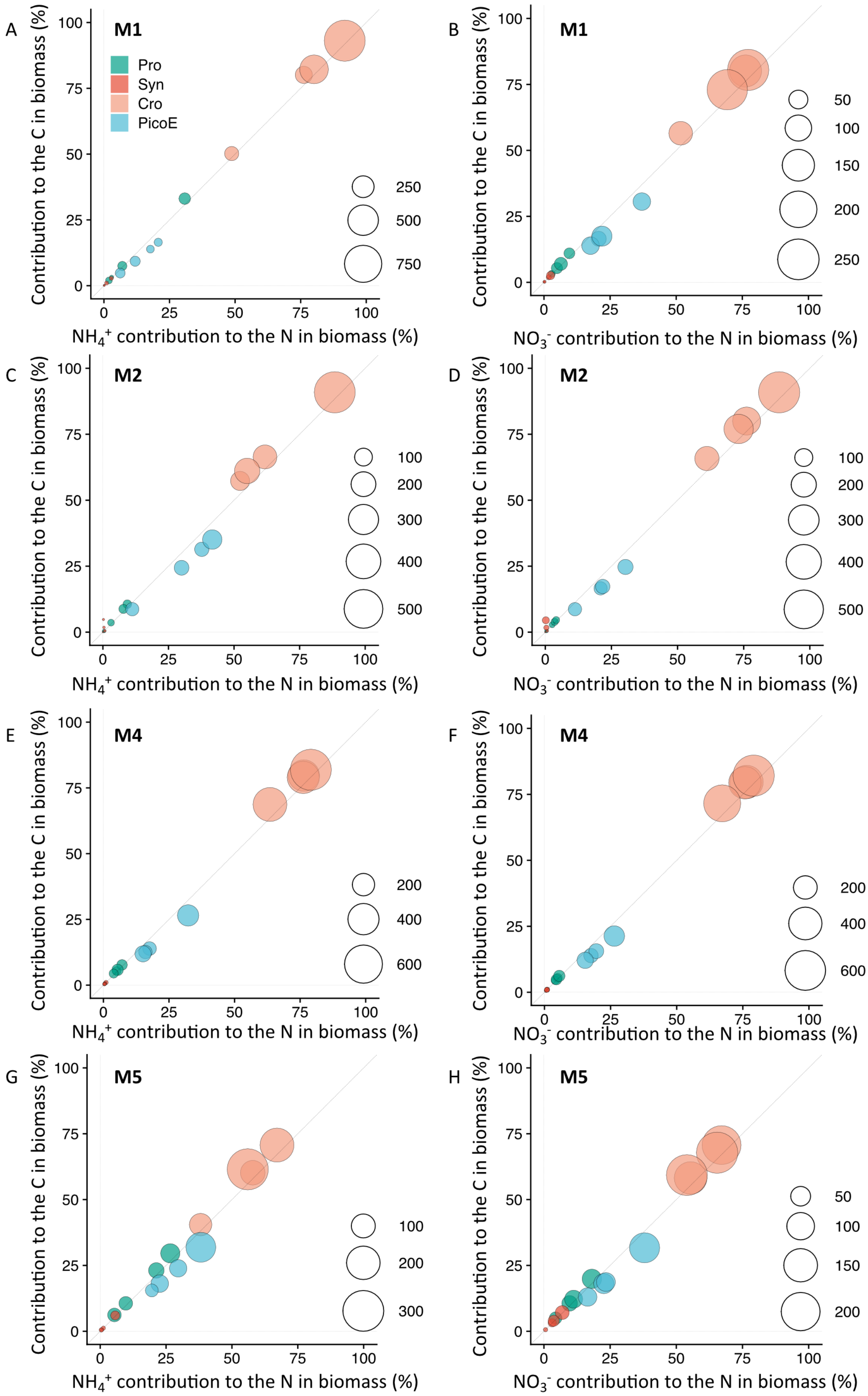
A
M1
Pro
Syn
Cro
PicoE
250
500
750
NH4+ contribution to the N in biomass (%)
Contribution to the C in biomass (%)
B
M1
50
100
150
200
250
NO3- contribution to the N in biomass (%)
C
M2
D
M2
E
M4
F
M4
G
M5
H
M5

Fig. S4 (A, C, E, G) Contribution to total carbon C in biomass as a function of the contribution of NH_4^+ - N biomass for each phytoplankton group. (B, D, F, H) Contribution to total carbon C in biomass as a function of the contribution of NO_3^- - N. The contributions of NH_4^+ - or NO_3^- - N were estimated from either NH_4^+ or NO_3^- enrichment. N biomass for each phytoplankton group. Each circle shows data from a different day, and the size of the dots represents the total C in biomass (nmol C L^{-1}). Pro; *Prochlorococcus*, Syn; *Synechococcus,* Cro; *Crocosphaera*, PicoE; pico-eukaryotes.

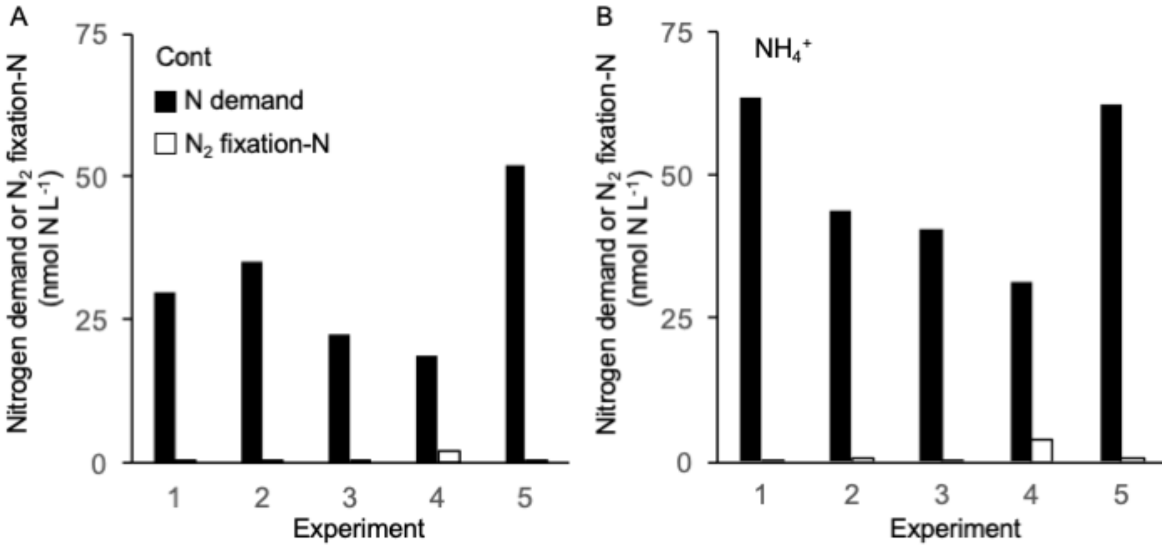

Fig. S5 Nitrogen demand and N derived from N_2 fixation in Control (A) and NH_4^+ treatment (B) for each experiment (M1-M5). Nitrogen demand is N in biomass in 3 days. N_2 fixation rate was estimated from the reported maximum cellular N_2 fixation rate 1.12 fmol N mol cell^{-1} day^{-1} (valued obtained in day 3 in Fe + N treatment of Fe3 (2)) and cell density.

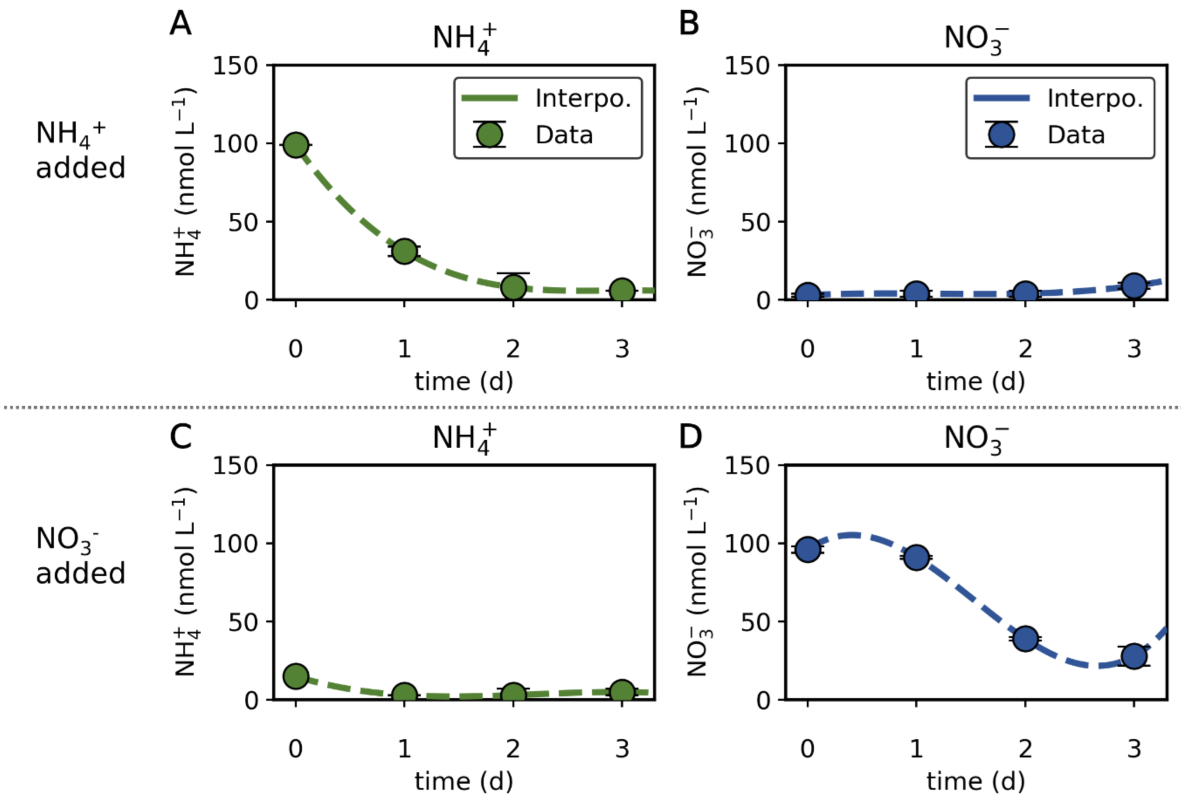

Fig. S6 Measured NH_4^+ and NO_3^- concentrations for NH_4^+ and NO_3^- added cases. Dashed lines show quadratic interpolation. Data are from experiment M3.

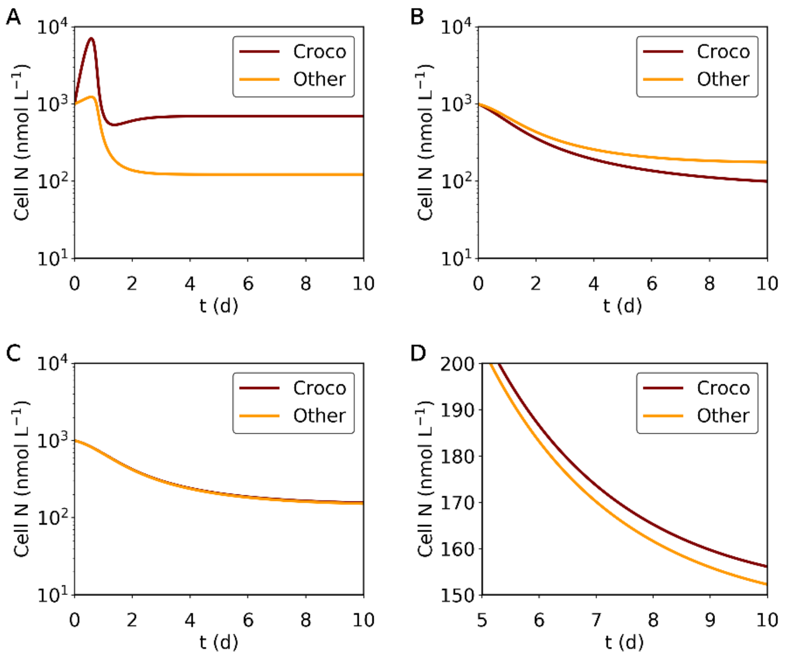

Fig. S7 Simulated transition of cellular N in a simple ecosystem model for three different scenarios. (A) The concentrations for NH_4^+ and NO_3^- are both 100 nmol L^{-1}. (B)(C) The concentrations for NH_4^+ and NO_3^- are both 1 nmol L^{-1}. In only (C) *Crocosphaera* may acquire N via N_2 fixation. (D) The same results as (C) but plotted in a linear scale and different axis ranges. Croco: *Crocosphaera*. Other: other phytoplankton. Parameters are based on NO_3^- added case.

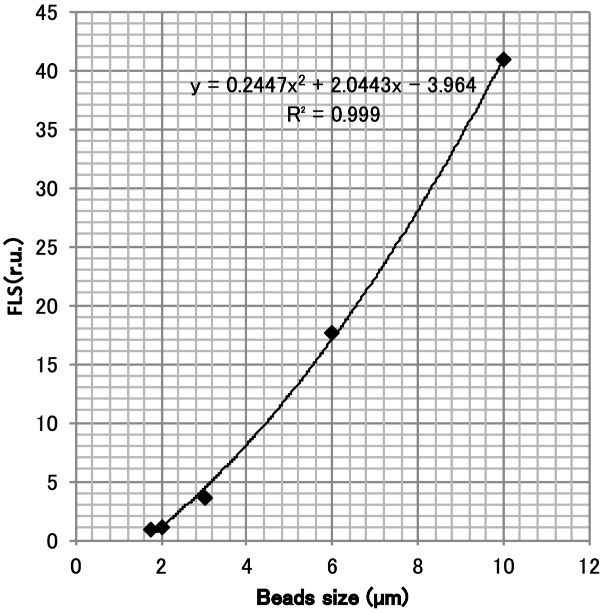

Fig. S8 The relationship between beads size (μm) and forward light scatter (FLS), which used to estimate phytoplankton cell size.

References

1. Inomura K, Masuda T, Gauglitz JM. 2019. Active nitrogen fixation by *Crocosphaera* expands their niche despite the presence of ammonium - A case study. Sci Rep 9:15064.
2. Masuda T, Inomura K, Mareš J, Kodama T, Shiozaki T, Matsui T, Suzuki K, Takeda S, Deutsch C, Prašil O, Furuya K. Pre print. Coexistence of dominant marine phytoplankton species sustained by nutrient specialization.
